# Training in Clinical Research in India

**DOI:** 10.4103/0970-0218.69289

**Published:** 2010-07

**Authors:** Sourabh Aggarwal, Harkirat Singh, Pranshu Bansal, Adarsh Goyal, Kalra Saminder Singh

**Affiliations:** University College of Medical Sciences, New Delhi, India. E-mail: drsourabh79@gmail.com; 1 All India Institute of Medical Sciences, New Delhi, India; 2 Vardhman Mahavir Medical College, New Delhi, India; 3 Government Medical College, Amritsar, India; 4 Dayanand Medical College, Ludhiana, Punjab, India

Sir,

The article “Training in Clinical Research in India: Potential and Challenges”(1) is an eye opener. It exposes the reasons for the lack of quality and quantity of human resources in the Indian scientific community in the field of clinical research activities. We, being medical students, would like to add our views on the same.

We believe that the medical curriculum needs to be revised especially in the wake of research-oriented teaching in the medical colleges. In India, as opposed to medical schools in developed countries like USA and UK, no stress is laid on the research activities and research experience. Though ICMR (Indian Council of Medical Research), through STS (Short-Term Studentship) and KVPY (Kishore Vaigyanik Protsahan Yojana) scholarship program by Department of Science and Technology, promote research activities at the undergraduate level, little efforts are done by the college or university management for the same. No emphasis is laid on the research interest and experience while selecting candidates for the postgraduation courses (MS or MD) in India. In contrast, in USA the research experience and papers published in journals are considered an important parameter by the Program Directors in conjunction with the good scores in USMLE (United States Medical Licensing Examination) to judge the credentials of the potential postgraduation candidates. As a result, the students spend considerable time in research activities during and after graduation to get into the better residency and fellowship programs and contribute significantly to clinical research. Though students in postgraduation in India are required to submit their thesis paper to qualify for degree, most do it as an obligation rather than out of interest.

We believe that increased exposure given to medical students during their undergraduation course can substantially increase their interest in this field and can provide a solution to the lacuna of manpower in the clinical research activities. Moreover, postgraduate programs similar to Masters of Public Health (MPH), MSc in Global Health Science, Health Economics Research, etc., which attract a lot of doctors in USA and UK should be promoted by the universities in India. As there is no paucity in variety of patients in India, clinical-research-oriented doctors can easily gather and analyze a large pool of data. It will not only contribute significantly to evidence-based medicine including increased knowledge of risk factors, presentations, progression, diagnosis, effective low-cost treatment, prevention strategies of diseases but will also play a significant role in pharmacovigilance. It will help to enhance our understanding about diseases with respect to indigenous epidemiologic data rather than relying on foreign research and data. Thus medical practitioners can not only provide effective health care to the patients but also contribute to the help in the evolution of medicine in a larger perspective.
